# How Reproducible Is the Data from Sysmex DI-60 in Leukopenic Samples?

**DOI:** 10.3390/diagnostics11122173

**Published:** 2021-11-23

**Authors:** Sumi Yoon, Mina Hur, Gun Hyuk Lee, Minjeong Nam, Hanah Kim

**Affiliations:** 1 Department of Laboratory Medicine, Chung-Ang University College of Medicine, Seoul 06973, Korea; ssumy17177@gmail.com; 2 Department of Laboratory Medicine, Konkuk University School of Medicine, Seoul 05030, Korea; leegunhyuk93@gmail.com (G.H.L.); md.hkim@gmail.com (H.K.); 3 Department of Laboratory Medicine, Korea University Anam Hospital, Seoul 02841, Korea; blueccoma@gmail.com

**Keywords:** leukopenic samples, precision, performance, Sysmex DI-60, digital morphology, white blood cell differentials

## Abstract

Digital morphology (DM) analyzers are widely applied in clinical practice. It is necessary to evaluate performances of DM analyzers by focusing on leukopenic samples. We evaluated the analytical performance, including precision, of a Sysmex DI-60 system (Sysmex, Kobe, Japan) on white blood cell (WBC) differentials in leukopenic samples. In a total of 40 peripheral blood smears divided into four groups according to WBC count (normal, mild, moderate, and severe leukopenia; each group *n* = 10), we evaluated precision of WBC preclassificaiton by DI-60. %coefficients of variation (%CVs) of precision varied for each sample and for each cell class; the fewer cells per slide, the higher %CV. The overall specificity and efficiency were high for all cell classes except plasma cells (95.9–99.9% and 90.0–99.4%, respectively). The largest absolute value of mean difference between DI-60 and manual count in each group was: 10.77, normal; 10.22, mild leukopenia; 19.09, moderate leukopenia; 47.74, severe leukopenia. This is the first study that evaluated the analytical performance of DI-60 on WBC differentials in leukopenic samples as the main subject. DI-60 showed significantly different performance depending on WBC count. DM analyzers should be evaluated separately in leukopenic samples, even if the overall performance was acceptable.

## 1. Introduction

Morphological analysis of peripheral blood smear (PBS) is mandatory for diagnosing hematologic diseases [1]. Following the complete blood count (CBC), PBS examination performed by light microscopy is the second most common test in hematology laboratories [2,3]. The white blood cell (WBC) differential on PBS is performed by the manual count, which two hematology experts count 200 cells each [4]. Although the manual count is still the gold standard method, it is time-/labor-intensive and subjective with large inter-/intra-observer variation and requires skilled observers who are trained continuously [1,5]. These disadvantages of manual count have led to an increased demand for developing automated analyzers for PBS examination [1].

In the present era of digital technology, digital morphology (DM) analyzers for PBS examination have become widely available in clinical practice [3]. According to the survey by the Institute for Quality Management in Healthcare (IQMH) [3], the most used DM analyzers used in clinical laboratories were CellaVision (CellaVision AB, Lund, Sweden) and Sysmex DI-60 (DI-60, Sysmex, Kobe, Japan) systems. Other DM analyzers include Vision Pro (West Medica, Perchtoldsdorf, Austria), EasyCell assistant (Medica, Bedford, MA, USA), Nextslide (Nextslide Imaging, LLC, Cleveland, OH, USA), and so on [1]. Since DM analyzers were first developed in the 1970s, a number of studies have been reported to evaluate the performance of DM analyzers [1,5,6,7,8,9,10,11,12,13,14,15,16,17,18,19,20].

The International Council for Standardization in Haematology (ICSH) has emphasized that all performance characteristics of DM analyzers used in clinical practice should be fully evaluated including the study of pathological samples [21]. Among pathological samples, the study of leukopenic samples is important because the performance of DM analyzers could vary, and the results in low values could be masked [21]. DM analyzers are highly affected by the quality of PBS and stain [2,16,21]. Internal Quality Control (IQC) and External Quality Assurance (EQA) are also valuable for monitoring of their performance [21,22].

It is necessary to evaluate the performance of DM analyzers on WBC differentials by focusing on leukopenic samples. Most of previous studies evaluated DM analyzers using pathological samples including some leukopenic samples [1,5,6,7,8,9,10,11,12,13,14,15,16,17,18,19,20]. To the best of our knowledge, however, there has been no study that analyzed leukopenic samples separately as the main subjects to fully evaluate the performance of DM analyzers, including precision [1,5,6,7,8,9,10,11,12,13,14,15,16,17,18,19,20]. In this study, we aimed to evaluate the performance of DI-60 on WBC differentials, especially precision according to the Clinical and Laboratory Standards Institute (CLSI) guidelines, using PBS of leukopenic samples. We also compared WBC preclassification and verification by DI-60 with reference WBC differentials by manual count.

## 2. Materials and Methods

### 2.1. Blood Samples

This study was conducted from January to April in 2021 in Konkuk University Medical Center (KUMC, Seoul, Korea). This study protocol was approved by the Institutional Review Board of KUMC (KUMC 2021-01-041 and 29 January 2021) before recruiting the first sample. Informed consent from subjects was waived because we used residual samples after performing the requested CBC and PBS examination. Venous whole blood samples were collected in a K_3_-EDTA-containing vacuette (Greiner Bio-One International GmbH, Frickenhausen, Germany). The CBC for WBC count were performed by XN-9000 (Sysmex) within 4 h after collecting samples. The slides of PBS were prepared and stained automatically using SP-10 slide maker/stainer (Sysmex) and Wright Giemsa (RAL Diagnostics, Bordeaux, France), respectively.

A total of 40 samples were collected and divided into four groups according to the WBC count: normal in number (4.0–10.0 × 10^9^/L, *n* = 10), mild leukopenia (2.0–4.0 × 10^9^/L, *n* = 10), moderate leukopenia (1.0–2.0 × 10^9^/L, *n* = 10), and severe leukopenia (<1.0 × 10^9^/L, *n* = 10). All samples were obtained from subjects (median age, 64 years; interquartile range [IQR], 33–76 years) who visited KUMC for diagnosing diseases or monitoring conditions: hematologic diseases including acute myeloid leukemia (*n* = 18), malignant lymphoma (*n* = 5), plasma cell myeloma (*n* = 4), myelodysplastic syndrome (*n* = 3), acute lymphoblastic leukemia (*n* = 1), aplastic anemia (*n* = 1), and chronic myeloid leukemia (*n* = 1); no hematologic diseases including bone marrow donor (*n* = 2), cardiac disease (*n* = 2), liver disease (*n* = 2), and breast cancer (*n* = 1).

### 2.2. WBC Differentials by Manual Count and DI-60

For reference WBC differentials, the manual count was performed according to the CLSI H20-A2 guidelines [4]. Two trained and experienced hematology experts scanned PBS at low magnification using light microscopy and counted 200 cells each on each slide at 200× magnification; an additional slide was processed if the counted cells on each slide were less than 200 cells [4]. The average values of the results obtained from two experts were used for evaluation; a third expert performed WBC differentials as an arbitrator only if the data was discrepant between the two experts [4].

DI-60 consists of a scanning microscope with two objectives (10× and 100×), intermediate optics switching (1.0× and 0.5×), a digital camera, and a computer system with the DI-60 remote review software (version 6.0) that acquires and preclassifies cells [12,15,23]. DI-60 can be integrated with the XN-9000/9100 or XN-3000/3100 (Sysmex) to create a fully automated hematology system that process CBC analysis, slide making/staining, and cell preclassification by digital scanning [12,23]. DI-60 can preclassify WBC into 18 classes: leukocytes (segmented and band neutrophils, lymphocytes, monocytes, eosinophils, basophils, blasts, promyelocytes, myelocytes, metamyelocytes, atypical lymphocytes, and plasma cells); non-leukocytes (nucleated red blood cells [nRBCs], smudge cells, artifacts, giant platelets, platelet clumps, and unidentified) [23]. The number of WBC that DI-60 preclassifies can be set by the user [15]. Considering DI-60 can preclassify WBC into non-leukocytes, we set up DI-60 to preclassify 210 cells. Verification of preclassified cells was performed by an expert. DI-60 can analyze RBC and platelet characteristics as well as WBC differentials [23]. In this study, we focused only on evaluating the performance of DI-60 on WBC differentials. WBC differentials using DI-60 were performed according to the manufacturer’s instructions.

### 2.3. Statistical Analysis

Data was expressed as median (IQR) or number (percentage, %). To evaluate precision, all samples were analyzed in five replicates per run, one run per day, for five days (5 × 5 experiment design for each sample) by adopting the CLSI EP15-A3 and EP15-Ed3-IG1 guidelines [24,25]. Repeatability and within-laboratory precision were evaluated by analysis of variance (ANOVA) and expressed as standard deviation (SD) and %coefficients of variation (%CV). Those were evaluated by dividing the cell classes into neutrophils (segmented and band forms), lymphocytes (including atypical lymphocytes), monocytes, eosinophils, basophils, immature granulocytes (IGs; promyelocytes, myelocytes, and metamyelocytes), blasts, plasma cells, nRBCs, and others (smudge cells, artifacts, giant platelets, platelet clumps, and unidentified). %CV were interpreted as follows: %CV ≤ 10%, excellent; %CV 10–20%, good; %CV 20–30%, acceptable; %CV > 30%, poor [26,27]. Each sample had a different cell count for each cell class and a different WBC differential; thus, the precision was evaluated for each sample.

The performances were evaluated using sensitivity, specificity, positive predictive value (PPV), negative predictive value (NPV), and efficiency of DI-60 preclassification on the basis of verification in a total of 40 samples. In addition, those were evaluated in each group according to the WBC count. Those were evaluated by dividing the cell classes into neutrophils, lymphocytes, monocytes, eosinophils, basophils, IGs, blasts, plasma cells, nRBCs, and others.

WBC preclassification and verification by DI-60 were compared with WBC differentials by manual count, respectively. Manual count was considered gold standard and reference for each comparison, appropriately. Wilcoxon test for paired samples, Bland-Altman plot, and Passing-Bablok regression analysis were used to compare DI-60 and manual count in total samples. In addition, Bland-Altman plot was applied to each group according to the WBC count. The mean difference using Bland-Altman plot was evaluated by dividing the cell classes into neutrophils, lymphocytes, monocytes, eosinophils, basophils, IGs, blasts, plasma cells, nRBCs, and others. The correlation using Passing-Bablok regression was evaluated by five WBCs (neutrophils, lymphocytes, monocytes, eosinophils, and basophils) only. Pearson’s correlation coefficients (r) with 95% confidence interval (CI) were obtained and interpreted as follows: <0.30, negligible; 0.30–0.50, low; 0.50–0.70, moderate; 0.70–0.90, high; 0.90–1.00, very high [28]. Statistical analyses were performed using Microsoft Excel Software (version 2016; Microsoft Corporation, Redmond, WA, USA) and MedCalc Statistical Software (version 20; MedCalc Software, Ostend, Belgium); two-sided *p* values less than 0.05 were considered statistically significant.

## 3. Results

The counted cells per slide by DI-60 and manual count are presented in Table 1. With the setting of 210 WBC counting, 222.5 cells (IQR, 79–231.5 cells) per slide were counted by DI-60 in a total of 40 samples, and less than 210 cells per slide in 15 samples (five moderate leukopenia and all 10 severe leukopenia). 200 cells (IQR, 133.5–200 cells) per slide were counted by manual count in total samples, and less than 200 cells per slide in 11 samples (one moderate leukopenia and all 10 severe leukopenia).

%CV of repeatability and within-laboratory precision varied across each sample and each cell class. %CV of repeatability and within-laboratory precision in total samples were excellent for neutrophils, good for lymphocytes and others, acceptable for monocytes, and poor for eosinophils, basophils, IGs, blasts, plasma cells, and nRBCs (Table 2). Those in each group according to the WBC count were presented in Table 2; the fewer cells per slide, the higher %CV.

The overall sensitivity was high for neutrophils, lymphocytes, basophils, blasts, and nRBCs (range, 86.5–95.8%), and relatively low for monocytes, eosinophils, IGs, and others (range, 52.6–66.6%). The overall specificity and efficiency were high for all cell classes except plasma cells (range, 95.9–99.9% and 90.0–99.4%, respectively). The sensitivity and efficiency of plasma cells were not available because all 10 plasma cells preclassified by DI-60 were verified as cells other than plasma cells. The sensitivity was variable in each group according to the WBC count; however, the specificity, PPV, NPV, and efficiency were similar to the overall results (Table 3).

**Figure 1:**
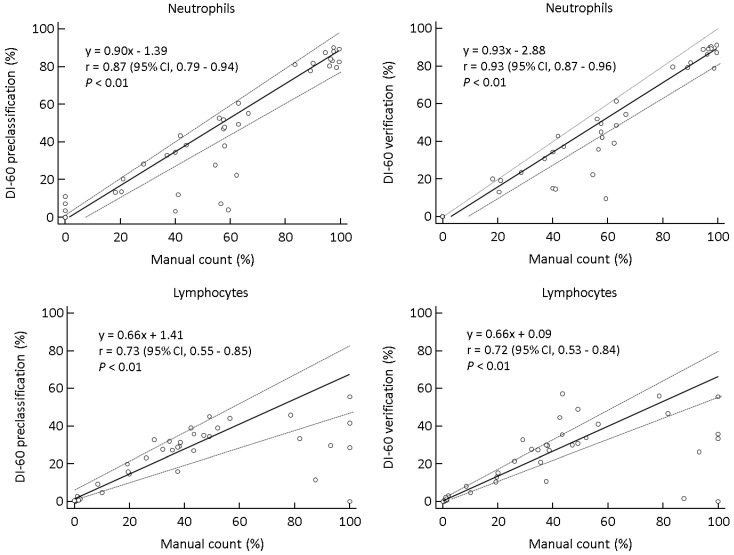

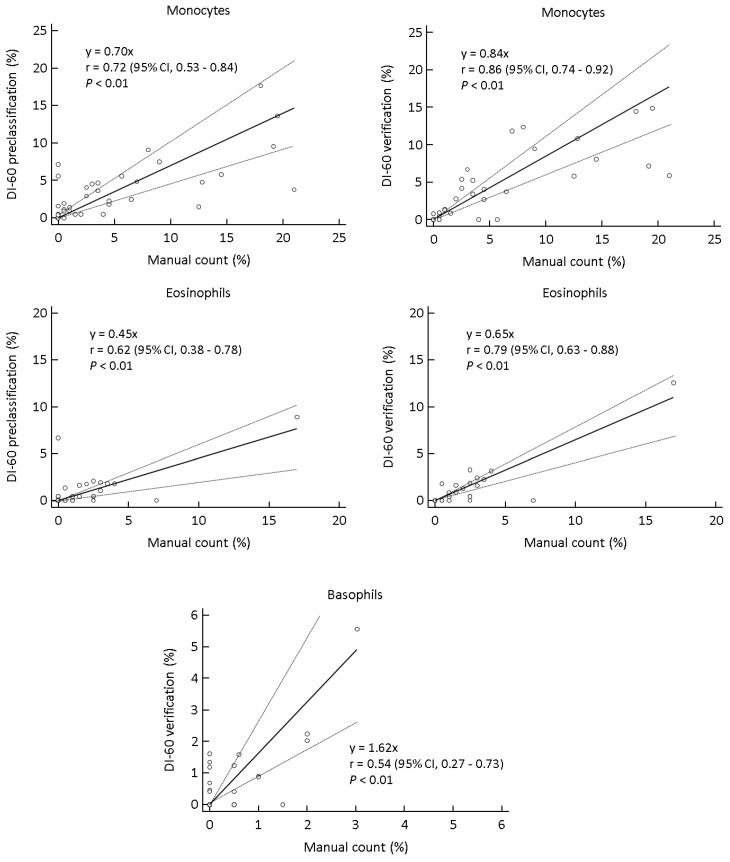
Comparison of five WBC differentials between DI-60 and manual count (*n* = 40). The data of basophils between DI-60 preclassification and manual count were not suitable for Passing-Bablok regression (r = 0.21, *p* = 0.19). Solid line, Passing-Bablok regression; dashed line, 95% CI line. Abbreviations: CI, confidence interval; WBC, white blood cells.

DI-60 preclassification and verification showed significant differences for basophils, IGs, blasts, nRBCs, and others (*p* < 0.01, respectively). DI-60 preclassification and manual count showed significant differences for all cell classes except for plasma cells (*p* = 0.10) and others that were not available because those were not counted by manual count. DI-60 verification and manual count showed significant differences for neutrophils, lymphocytes, and eosinophils (*p* < 0.01, respectively). In total samples, the absolute values of mean differences between DI-60 preclassification and manual count ranged from 0.30 to 16.28; those between DI-60 verification and manual count ranged from 0.00 to 16.36. According to the WBC count, the absolute values of mean differences between DI-60 and manual count ranged from 0.00 to 10.77 in the group with normal WBC count; 0.00 to 10.22 in the group with mild leukopenia; 0.00 to 19.09 in group with moderate leukopenia; 0.00 to 47.74 in the group with severe leukopenia (Table 4). DI-60 preclassification and manual count showed high correlations for neutrophils (r = 0.87), lymphocytes (r = 0.73), and monocytes (r = 0.72); moderate correlations for eosinophils (r = 0.62). The data of basophils were not suitable for Passing-Bablok regression (r = 0.21, *p* = 0.19). After verification, the correlations between DI-60 and manual count were improved for all cell class (r = 0.54 to 0.93) except lymphocytes (r = 0.72) (Figure 1).

## 4. Discussion and Conclusions

According to the ICSH guidelines [21], mean difference or Bland–Altman plots should be evaluated in leukopenic, anemic or thrombocytopenic samples. In our previous study that evaluated a DM analyzer Vision Pro on WBC differentials, we found that the mean differences between the Vision Pro and manual count results were larger when analyzing only leukopenic samples than total samples [20]. Based on our previous study, we questioned and evaluated the analytical performance of DI-60 on WBC differentials in leukopenic samples, especially focusing on the precision. We also compared WBC differentials by DI-60 and manual count in leukopenic samples.

DI-60 counted nearly 210 cells in most samples, except severe leukopenic samples; similarly, manual count did not count 200 cells in severe leukopenic samples (Table 1). The precision of DI-60 on WBC differentials differed between cell classes even in the same sample. DI-60 tended to be more imprecise with fewer WBCs in the sample (Table 2). Like other studies where automated CBC hematology analyzers showed less precise WBC differentials in relatively few cells, a DM analyzer DI-60 showed similar results [29,30,31,32]. DI-60 on WBC preclassification showed high efficiency with verification in total samples; even in each group according to the WBC count (Table 3). These results are similar to previous studies that evaluated the performance of DI-60, DM96, and Vision Pro [9,15,20]. However, DI-60 tended to show lower sensitivity with fewer WBCs in the sample for neutrophils, lymphocytes, and monocytes. DI-60 showed low PPV for basophils, IGs, blasts, and nRBCs on WBC preclassification compared with verification. These results indicate that many basophils, IGs, blasts, and nRBCs preclassified by DI-60 are misclassified. WBC verification is especially required when these cells are preclassified a lot. Our results are in line with the ICSH review and recommendations that hematology experts should perform cell verification and slide review [1].

The largest absolute value of mean differences between DI-60 and manual count tended to be larger with fewer WBCs in the sample. WBC differentials by DI-60 compared with manual count did not show acceptable differences, especially for neutrophils and lymphocytes (Table 4). These results are similar to our previous study that Vision Pro and manual count showed larger mean differences in leukopenic samples than total samples [20]; however, in this study, the differences were much larger than in previous study. This may be due to the higher proportion of severe leukopenic samples in this study. Comparing five WBC differentials by DI-60 with those by manual count, the correlations were moderate to high except basophils and improved after verification (Figure 1). Several studies have reported that basophil showed a larger variation and a lower correlation because it is a small number of cells and its detection is highly affected depending on where the PBS is read [20,33,34]. Similarly in this study, basophils preclassified by DI-60 were unable to perform Passing-Bablok regression.

Microscopic review is mandatory for leukopenic samples in routine hematology practice [35]. The strength of this study is that it provides fundamental data on the performance of DM analyzers in leukopenic samples, which can be a cornerstone for further studies. On the other hand, this study also has several limitations. First, the number of samples in each group according to the WBC count was small. However, a total of 1000 times of DI-60 preclassification were performed for evaluating precision (5 × 5 × 40 samples), which also required tedious and labor-intensive manual workload. It is clinically relevant to evaluate DM analyzers separately in leukopenic samples. Nevertheless, considering that the number of samples is limited, it is reasonable to consider our findings as preliminary. Our findings could be supported by further studies with a large number of leukopenic samples. In addition, the CLSI H20-A2 guidelines recommend additional slides for leukopenic samples, but it was practically difficult to prepare a larger number of slides for all severe leukopenic samples [4]. There is a limit to applying the guidelines to laboratory practice as they are, and it is a pitfall of the guidelines. Second, we evaluated DI-60 focusing only on WBC differentials. DM analyzers should be evaluated not only for WBC differentials but also for RBC and platelet characteristics even in anemic and/or thrombocytopenic samples [21]. Third, DM analyzers are highly affected by the smear and staining quality of the blood films, and in a previous study, DI-60 showed various analytical performances depending on the staining methods [16,21]. An acceptable blood film is required for optimal microscopic review, and quality control of DM analyzers should be assessed at regular intervals using IQC blood films, [1,4,21]. Further studies would be needed to focus on the quality control issue in leukopenic samples. Last, although we evaluated within-laboratory precision, we could not evaluate interlaboratory precision. Evaluation of interlaboratory precision is important to ensure reliable and comparable results between DM analyzers performed at different laboratories [33,36]. A standardized approach and protocol are also necessary for evaluating precision of WBC differentials in both DM analyzers and manual count [4,21]. In addition to IQC, laboratories should assess the quality control through EQA programs [1,21,22,37].

In conclusion, DI-60 on WBC differentials showed significantly different performances according to the WBC count in leukopenic samples. This study suggests that DM analyzers should be evaluated separately in leukopenic samples, even if the overall performance would be acceptable in total samples. In particular, laboratories with many leukopenic samples need to deal with such samples separately in terms of establishing slide review criteria and assessing quality control to guarantee more efficient application of DM analyzers and improve hematology workflow.

## Figures and Tables

**Table 1:** Study population and counted cells per slide of samples by DI-60 and manual count.

Sample	WBC Count (×10^9^/L), Median (IQR)	Counted Cells per Slide by DI-60, Median (IQR)	Counted Cells per Slide by Manual Count, Median (IQR)
Total (*n* = 40)	2.215 (0.770–4.565)	222.5 (79–231.5)	200 (133.5–200)
WBC count ^a^			
Normal in number (*n* = 10)	7.010 (5.880–7.590)	227.5 (222–238)	200 (200–200)
Mild leukopenia (*n* = 10)	2.855 (2.640–3.400)	228.5 (223–242)	200 (200–200)
Moderate leukopenia (*n* = 10)	1.470 (1.130–1.890)	216 (187–227)	200 (200–200)
Severe leukopenia (*n* = 10)	0.075 (0.060–0.180)	14.5 (12–18)	15.5 (6–46)
Diagnosis			
AML (*n* = 18)	1.420 (0.150–1.970)	208 (15–225)	200 (33–200)
Malignant lymphoma (*n* = 5)	2.590 (1.120–2.830)	226 (171.8–241.3)	200 (191.8–200)
Others ^b^ (*n* = 17)	NA	NA	NA

^a^ Normal, 4.0–10.0 × 10^9^/L; mild, 2.0–4.0 × 10^9^/L; moderate, 1.0–2.0 × 10^9^/L; severe, <1.0 × 10^9^/L. ^b^ Others include plasma cell myeloma, myelodysplastic syndrome, acute lymphoblastic leukemia, aplastic anemia, chronic myeloid leukemia, bone marrow donor, cardiac disease, liver disease, and breast cancer; the data in this group (with *n* < 5 samples each disease) is not expressed as median (IQR). Abbreviations: AML, acute myeloid leukemia; IQR, interquartile range; NA, not available; WBC, white blood cell.

**Table 2:** Precision of DI-60 preclassification (5 × 5 experiment design for each sample).

Cell Class	Number of Cells, Median (IQR)	Mean Value of Cells per Slide, Median (IQR)	Repeatability		Within-Laboratory Precision	
			SD, Median (IQR)	%CV, Median (IQR)	SD, Median (IQR)	%CV, Median (IQR)
**Total Samples (*n* = 40)**						
Neutrophils	2325.5 (100–4269)	100.2 (6.4–178.4)	2.1 (1.5–3.3)	3.3 (1.9–26.3)	2.2 (1.6–3.5)	3.9 (2.0–26.3)
Lymphocytes	357 (104.5–1633)	16.7 (4.5–66.5)	2.2 (1.1–3.1)	16.3 (4.8–32.6)	2.4 (1.1–3.4)	17.7 (5.7–32.8)
Monocytes	56 (20.5–237)	3.5 (1.5–10.4)	1.0 (0.6–1.6)	22.3 (12.8–40.4)	1.0 (0.6–1.7)	25.9 (14.2–42.4)
Eosinophils	7.5 (0–42)	1.1 (0.3–3.0)	0.6 (0.4–0.7)	52.8 (33.3–181.2)	0.7 (0.4–0.8)	60.9 (35.9–190.1)
Basophils	88.5 (30–137.5)	3.6 (1.2–5.5)	1.2 (0.8–1.4)	31.9 (26.6–70.3)	1.2 (0.9–1.5)	33.6 (28.8–81.6)
IGs	62.5 (13–126)	3.0 (0.9–5.5)	1.1 (0.7–1.8)	45.2 (30.0–75.0)	1.2 (0.8–2.0)	47.1 (32.0–82.3)
Blasts	16.5 (1–49)	1.0 (0.6–2.1)	0.7 (0.5–0.9)	60.8 (36.3–121.4)	0.7 (0.5–1.1)	72.5 (37.5–133.8)
Plasma cells	1 (0–9.5)	0.4 (0.1–0.7)	0.4 (0.2–0.5)	144.3 (90.5–318.1)	0.5 (0.3–0.5)	153.1 (100.1–353.6)
nRBCs	24 (11–43.5)	1.0 (0.5–1.8)	0.9 (0.6–1.2)	88.4 (54.4–143.3)	0.9 (0.6–1.3)	95.2 (59.1–153.2)
Others	548.5 (281–830.5)	22.0 (11.3–33.2)	3.9 (2.9–5.9)	17.4 (13.5–28.6)	4.2 (3.2–6.3)	19.3 (14.8–30.6)
**Normal in Number (*n* = 10)**						
Neutrophils	3149 (2813–4538)	126.0 (112.5–181.5)	2.5 (2.1–3.4)	2.0 (1.2–3.0)	2.6 (2.2–3.5)	2.1 (1.2–3.1)
Lymphocytes	1445 (135–1805)	57.8 (5.4–72.2)	2.3 (1.1–3.4)	5.8 (4.2–19.4)	2.8 (1.1–4.0)	7.0 (4.7–19.4)
Monocytes	173.5 (50–211)	7.0 (2.0–8.4)	1.1 (0.7–1.6)	23.7 (14.4–35.4)	1.2 (0.7–1.6)	23.7 (14.6–35.4)
Eosinophils	12 (1–49)	0.6 (0.1–2.3)	0.6 (0.3–0.8)	100.2 (38.0–341.5)	0.6 (0.3–0.9)	111.8 (38.2–341.5)
Basophils	121.5 (73–138)	4.9 (2.9–5.5)	1.4 (1.1–1.8)	33.7 (29.8–45.6)	1.5 (1.1–1.9)	36.0 (29.8–45.6)
IGs	62.5 (22–88)	2.5 (0.9–3.5)	1.3 (0.5–2.1)	52.6 (34.0–89.5)	1.4 (0.7–2.1)	64.0 (47.1–89.5)
Blasts	12 (1–22)	0.7 (0.3–1.4)	0.6 (0.3–1.0)	121.4 (58.3–202.5)	0.6 (0.3–1.0)	121.4 (61.5–211.9)
Plasma cells	0 (0–1)	0.1 (0.0–0.6)	0.3 (0.2–0.4)	288.7 (114.1–447.2)	0.3 (0.2–0.5)	288.7 (120.0–447.2)
nRBCs	11 (9–19)	0.4 (0.4–0.8)	0.6 (0.5–0.8)	133.7 (113.9–180.0)	0.7 (0.5–0.9)	144.7 (120.3–180.0)
Others	547.5 (471–805)	21.9 (18.8–32.2)	3.0 (2.8–3.5)	13.9 (11.2–17.3)	3.8 (3.1–4.7)	15.4 (13.9–19.8)
**Mild Leukopenia (*n* = 10)**						
Neutrophils	2167 (1653–3442)	86.7 (66.1–137.7)	2.4 (1.7–3.3)	2.9 (1.8–4.2)	2.4 (1.9–3.7)	3.2 (2.0–4.2)
Lymphocytes	1336.5 (760–2370)	53.5 (30.4–94.8)	3.0 (2.1–3.2)	5.6 (3.4–9.8)	3.1 (2.5–3.8)	6.4 (3.4–10.5)
Monocytes	247 (143–294)	9.9 (5.7–11.8)	1.5 (0.7–1.8)	14.1 (11.6–19.4)	1.6 (0.7–2.1)	14.8 (11.9–23.2)
Eosinophils	43.5 (2–119)	2.1 (1.1–5.0)	0.7 (0.3–1.1)	45.2 (16.1–126.3)	0.8 (0.5–1.2)	45.2 (17.1–130.0)
Basophils	95.5 (60–156)	3.9 (2.4–6.2)	1.1 (0.9–1.5)	27.2 (25.7–31.3)	1.2 (0.9–1.5)	27.6 (25.7–31.3)
IGs	176 (54–260)	7.1 (2.2–10.4)	1.5 (1.1–2.1)	27.1 (20.0–32.1)	1.6 (1.1–2.2)	32.5 (20.1–39.6)
Blasts	64.5 (32–252)	2.6 (1.3–10.1)	0.9 (0.7–1.4)	41.3 (16.2–56.7)	1.0 (0.7–1.6)	43.3 (16.8–56.7)
Plasma cells	4.5 (1–9)	0.3 (0.1–0.5)	0.3 (0.2–0.5)	148.7 (114.0–284.9)	0.4 (0.3–0.5)	170.7 (122.6–316.3)
nRBCs	18.5 (14–41)	0.8 (0.6–1.7)	0.9 (0.7–1.1)	88.4 (69.0–130.1)	0.9 (0.7–1.2)	95.2 (76.1–130.1)
Others	641.5 (394–888)	25.7 (15.8–35.5)	4.1 (3.1–4.9)	16.8 (10.7–18.2)	4.1 (3.3–4.9)	17.6 (11.2–18.2)
**Moderate Leukopenia (*n* = 10)**						
Neutrophils	3353.5 (764–4562)	134.2 (30.6–182.5)	2.4 (1.5–5.6)	3.9 (2.0–13.9)	3.0 (1.6–5.6)	4.5 (2.1–13.9)
Lymphocytes	55 (33–1027)	2.2 (1.3–41.1)	0.7 (0.6–2.9)	34.0 (8.1–46.7)	0.8 (0.6–2.9)	34.8 (8.1–47.9)
Monocytes	51.5 (25–283)	2.1 (1.0–11.3)	0.9 (0.5–1.1)	28.8 (17.3–61.9)	0.9 (0.5–1.2)	29.8 (19.5–66.9)
Eosinophils	13.5 (6–34)	1.0 (0.4–2.2)	0.6 (0.5–0.7)	75.0 (35.6–149.5)	0.7 (0.5–0.7)	80.9 (37.1–158.4)
Basophils	107 (60–139)	4.3 (2.4–5.6)	1.3 (0.9–1.4)	28.5 (24.6–36.8)	1.4 (0.9–1.5)	32.9 (26.8–42.5)
IGs	90 (55–119)	3.6 (2.2–4.8)	1.4 (1.1–1.8)	37.9 (30.2–57.0)	1.5 (1.1–2.0)	38.4 (30.2–72.0)
Blasts	16 (12–48)	0.6 (0.6–1.9)	0.6 (0.6–0.8)	69.9 (44.7–119.1)	0.7 (0.6–0.9)	77.8 (52.3–130.2)
Plasma cells	0.5 (0–21)	0.8 (0.4–1.1)	0.5 (0.4–0.8)	95.2 (57.4–198.6)	0.6 (0.4–0.9)	109.1 (63.2–211.6)
nRBCs	48 (38–107)	2.0 (1.5–4.3)	1.4 (1.0–1.9)	60.6 (39.7–75.7)	1.4 (1.1–1.9)	60.6 (39.7–83.3)
Others	736.5 (549–1518)	29.5 (22.0–60.7)	6.8 (3.9–9.1)	18.9 (12.8–27.7)	7.5 (3.9–12.0)	19.5 (14.5–28.9)
**Severe Leukopenia (*n* = 10)**						
Neutrophils	27 (18–51)	1.6 (0.9–3.0)	0.8 (0.7–1.4)	57.4 (33.8–85.7)	0.8 (0.7–1.4)	57.4 (34.9–85.7)
Lymphocytes	141 (111–185)	5.9 (4.7–8.5)	1.4 (1.3–2.7)	28.9 (17.9–32.0)	1.5 (1.3–2.7)	28.9 (18.5–32.6)
Monocytes	3 (0–20)	0.8 (0.5–1.0)	0.4 (0.4–0.6)	66.3 (33.1–89.4)	0.5 (0.5–0.6)	75.3 (48.8–106.3)
Eosinophils	0	NA	NA	NA	NA	NA
Basophils	23.5 (9–52)	1.1 (0.4–2.5)	0.8 (0.6–1.1)	100.2 (45.5–147.8)	0.8 (0.6–1.2)	102.9 (51.5–147.8)
IGs	1.5 (0–22)	0.6 (0.0–1.1)	0.6 (0.2–0.7)	133.6 (59.0–463.4)	0.6 (0.2–0.9)	149.0 (71.4–463.4)
Blasts	0 (0–24)	1.0 (0.5–1.5)	0.4 (0.2–1.1)	66.2 (39.9–286.7)	0.4 (0.2–0.6)	75.0 (46.7–288.8)
Plasma cells	0.5 (0–10)	0.4 (0.1–0.5)	0.5 (0.3–0.6)	158.1 (117.0–390.2)	0.5 (0.3–0.6)	158.1 (117.0–390.2)
nRBCs	24 (10–43)	1.0 (0.4–1.7)	0.9 (0.6–1.2)	104.0 (59.9–158.1)	0.9 (0.6–1.4)	107.0 (66.2–162.0)
Others	154 (88–278)	6.2 (3.5–11.1)	3.2 (1.7–5.7)	44.4 (29.4–60.5)	3.3 (1.8–5.7)	48.0 (32.3–68.9)

>Abbreviations: %CV, %coefficients of variation; IGs, immature granulocytes; IQR, interquartile range; NA, not available; nRBCs, nucleated red blood cells; SD, standard deviation.

**Table 3:** Performance of WBC preclassification by DI-60 on the basis of verification.

Cell Class	Number of Cells	Sensitivity (%, 95% CI)	Specificity (%, 95% CI)	Positive Predictive Value (%, 95% CI)	Negative Predictive Value (%, 95% CI)	Efficiency (%, 95% CI)
**Total Samples (*n* = 40)**						
Neutrophils	3612	95.8 (95.1–96.4)	96.6 (95.9–97.2)	97.0 (96.4–97.5)	95.2 (94.5–95.9)	96.1 (95.7–96.6)
Lymphocytes	1347	86.5 (84.5–88.3)	95.9 (95.3–96.4)	83.0 (81.1–84.7)	96.8 (96.4–97.2)	94.1 (93.5–94.7)
Monocytes	249	60.0 (54.4–65.5)	99.1 (98.8–99.3)	75.9 (70.7–80.5)	98.1 (97.8–98.3)	97.3 (96.9–97.7)
Eosinophils	59	60.0 (48.4–70.8)	99.8 (99.7–99.9)	81.4 (70.2–89.0)	99.5 (99.4–99.6)	99.4 (99.2–99.5)
Basophils	139	91.2 (76.3–98.1)	98.4 (98.1–98.7)	22.3 (18.8–26.2)	99.9 (99.8–100.0)	98.4 (98.1–98.7)
IGs	142	52.6 (39.0–66.0)	98.4 (98.0–98.6)	21.1 (16.5–26.7)	99.6 (99.5–99.7)	98.0 (97.6–98.3)
Blasts	80	95.8 (78.9–99.9)	99.2 (98.9–99.4)	28.8 (23.5–34.6)	99.9 (99.9–100.0)	99.2 (98.9–99.4)
Plasma cells	10	NA	99.9 (99.7–99.9)	0.0	100.0	NA
nRBCs	75	86.7 (59.5–98.3)	99.1 (98.8–99.3)	17.3 (13.2–22.4)	99.9 (99.8–100.0)	99.1 (98.8–99.3)
Others	1146	66.6 (64.1–69.1)	95.9 (95.4–96.4)	80.5 (78.3–82.5)	91.9 (91.3–92.5)	90.0 (89.3–90.7)
**Normal in Number (*n* = 10)**						
Neutrophils	1435	98.3 (97.5–98.9)	96.3 (94.8–97.5)	97. (96.9–98.4)	97.2 (95.9–98.1)	97.6 (96.8–98.2)
Lymphocytes	450	91.1 (87.9–93.6)	96.1 (95.2–96.9)	83.8 (80.5–86.6)	98.0 (97.3–98.5)	95.2 (94.3–96.0)
Monocytes	62	65.2 (54.3–75.0)	99.8 (99.5–100.0)	93.5 (84.3–97.5)	98.6 (98.2–99.0)	98.5 (97.9–99.0)
Eosinophils	11	69.2 (38.6–90.9)	99.9 (99.7–100.0)	81.8 (51.8–95.0)	99.8 (99.6–99.9)	99.7 (99.4–99.9)
Basophils	41	80.0 (44.4–97.5)	98.6 (98.0–99.0)	19.5 (13.3–27.7)	99.9 (99.7–100.0)	98.5 (97.9–98.9)
IGs	24	NA	99.0 (98.4–99.3)	0.0	100.0	NA
Blasts	7	NA	99.7 (99.4–99.9)	0.0	100.0	NA
Plasma cells	1	NA	99.9 (99.8–100.0)	0.0	100.0	NA
nRBCs	9	NA	99.6 (99.3–99.8)	0.0	100.0	NA
Others	257	64.0 (58.6–69.0)	98.1 (97.4–98.7)	85.6 (81.1–89.2)	93.9 (93.1–94.7)	93.0 (91.9–94.0)
**Mild Leukopenia (*n* = 10)**						
Neutrophils	1032	97.6 (96.5–98.5)	96.2 (95.1–97.2)	95.1 (93.6–96.2)	98.2 (97.3–98.8)	96.8 (96.0–97.5)
Lymphocytes	570	88.8 (85.9–91.3)	95.6 (94.6–96.5)	86.1 (83.3–88.5)	96.5 (95.6–97.2)	94.0 (93.0–94.9)
Monocytes	116	63.6 (55.9–70.8)	99.7 (99.4–99.9)	94.8 (89.1–97.6)	97.2 (96.6–97.7)	97.1 (96.3–97.7)
Eosinophils	39	57.1 (43.2–70.3)	99.7 (99.4–99.9)	82.1 (67.8–90.8)	99.0 (98.6–99.2)	98.7 (98.1–99.1)
Basophils	39	93.3 (68.1–99.8)	98.9 (98.4–99.3)	35.9 (27.0–45.8)	99.9 (99.7–100.0)	98.9 (98.4–99.3)
IGs	65	50.0 (32.9–67.1)	98.0 (97.3–98.5)	27.7 (19.9–37.1)	99.2 (98.9–99.4)	97.2 (96.5–97.9)
Blasts	62	95.8 (78.9–99.9)	98.3 (97.7–98.8)	37.1 (29.9–44.9)	99.9 (99.7–100.0)	98.3 (97.7–98.8)
Plasma cells	3	NA	99.9 (99.6–100.0)	0.0	100.0	NA
nRBCs	21	92.3 (64.0–99.8)	99.6 (99.3–99.8)	57.1 (40.5–72.3)	99.9 (99.7–100.0)	99.6 (99.2–99.8)
Others	408	68.8 (64.4–72.9)	95.8 (94.8–96.7)	80.9 (77.2–84.1)	92.3 (91.3–93.2)	90.3 (89.1–91.5)
**Moderate Leukopenia (*n* = 10)**						
Neutrophils	1125	91.8 (90.1–93.3)	97.7 (96.3–98.6)	98.4 (97.5–99.0)	88.4 (86.3–90.2)	94.1 (93.0–95.1)
Lymphocytes	266	75.7 (70.2–80.8)	96.3 (95.3–97.2)	76.3 (71.5–80.6)	96.2 (95.3–97.0)	93.5 (92.4–94.6)
Monocytes	67	42.9 (28.8–57.8)	97.6 (96.8–98.2)	31.3 (22.9–41.3)	98.5 (98.1–98.8)	96.3 (95.3–97.1)
Eosinophils	8	63.6 (30.8–89.1)	99.9 (99.7–100.0)	87.5 (48.4–98.1)	99.8 (99.6–99.9)	99.7 (99.4–99.9)
Basophils	44	100.0 (63.1–100.0)	98.2 (97.5–98.7)	18.2 (13.9–23.5)	100.0	98.2 (97.5–98.7)
IGs	47	57.1 (34.0–78.2)	98.2 (97.5–98.8)	25.5 (17.3–36.0)	99.5 (99.2–99.7)	97.8 (97.0–98.4)
Blasts	10	NA	99.5 (99.1–99.8)	0.0	100.0	NA
Plasma cells	4	NA	99.8 (99.5–99.9)	0.0	100.0	NA
nRBCs	29	50.0 (1.3–98.7)	98.6 (98.0–99.1)	3.4 (0.8–13.0)	99.9 (99.8–100.0)	98.5 (97.9–99.0)
Others	378	66.8 (62.1–71.4)	93.5 (92.1–94.7)	73.0 (68.9–76.8)	91.4 (90.3–92.5)	87.9 (86.4–89.3)
**Severe Leukopenia (*n* = 10)**						
Neutrophils	20	57.9 (33.5–79.7)	95.7 (92.0–98.0)	55.0 (36.7–72.0)	96.2 (93.7–97.7)	92.6 (88.4–95.6)
Lymphocytes	61	81.0 (68.6–90.1)	91.8 (86.6–95.5)	77.0 (66.7–84.9)	93.5 (89.3–96.1)	89.1 (84.3–92.8)
Monocytes	4	0.0 (0.0–60.2)	98.2 (95.5–99.5)	0.0	98.2 (98.2–98.3)	96.5 (93.2–98.5)
Eosinophils	1	NA	99.6 (97.6–100.0)	0.0	100.0	NA
Basophils	15	100.0 (2.5–100.0)	93.9 (89.9–96.6)	6.7 (4.1–10.6)	100.0	93.9 (90.0–96.6)
IGs	6	NA	97.4 (94.4–99.0)	0.0	100.0	NA
Blasts	1	NA	99.6 (97.6–100.0)	0.0	100.0	NA
Plasma cells	2	NA	99.1 (96.9–99.9)	0.0	100.0	NA
nRBCs	16	NA	93.0 (88.9–96.0)	0.0	100.0	NA
Others	103	65.3 (57.0–73.0)	91.5 (83.2–96.5)	93.2 (87.0–96.6)	59.5 (53.8–65.0)	74.7 (68.5–80.2)

Abbreviations: CI, confidence interval; IGs, immature granulocytes; NA, not available; nRBCs, nucleated red blood cells; WBC, white blood cells.

**Table 4:** Comparison of WBC differentials by DI-60 and manual count.

	Number of Cells			Mean Difference (%, 95% CI)		
	Preclassification	Verification	Manual Count	Preclassification vs. Verification	Preclassification vs. Manual Count	Verification vs. Manual Count
**Total Samples (*n* = 40)**						
Neutrophils	3612	3657	4046	−1.30 (−3.45 to 0.85)	−11.34 (−15.91 to −6.77)	−10.04 (−13.38 to −6.70)
Lymphocytes	1347	1293	1663	0.08 (−1.83 to 1.99)	−16.28 (−24.37 to −8.19)	−16.36 (−24.67 to −8.04)
Monocytes	249	315	340	−0.30 (−1.16 to 0.56)	−1.41 (−2.82 to −0.01)	−1.12 (−2.35 to 0.12)
Eosinophils	59	80	113	−1.68 (−2.86 to −0.50)	−0.70 (−1.38 to −0.02)	−0.63 (−1.08 to −0.17)
Basophils	139	34	22	2.55 (1.54 to 3.56)	2.72 (1.69 to 3.75)	0.17 (−0.04 to 0.38)
IGs	142	57	46	1.39 (0.71 to 2.07)	1.44 (0.76 to 2.12)	0.05 (−0.17 to 0.28)
Blasts	80	24	21	0.62 (0.29 to 0.95)	0.64 (0.31 to 0.97)	0.02 (−0.02 to 0.05)
Plasma cells	10	0	0	0.30 (−0.07 to 0.67)	0.30 (−0.07 to 0.67)	0.00 (0.00 to 0.00)
nRBCs	75	15	3	2.91 (1.11 to 4.71)	3.04 (1.25 to 4.83)	0.13 (−0.06 to 0.32)
Others	1146	1384	NA	−6.19 (−10.42 to −2.0)	NA	NA
**Normal in Number (*n* = 10)**						
Neutrophils	1435	1427	1456	0.47 (−1.13 to 2.07)	−10.31 (−13.56 to −7.05)	−10.77 (−13.99 to −7.56)
Lymphocytes	450	414	447	1.59 (−0.01 to 3.19)	−2.73 (−6.34 to 0.87)	−4.32 (−8.97 to 0.32)
Monocytes	62	89	60	−1.21 (−2.06 to −0.36)	−0.30 (−1.64 to 1.04)	0.91 (−0.74 to 2.56)
Eosinophils	11	13	26	−1.12 (−2.43 to 0.20)	−0.81 (−1.55 to −0.06)	−0.68 (−1.24 to −0.12)
Basophils	41	10	10	1.35 (0.74 to 1.97)	1.29 (0.72 to 1.85)	−0.07 (−0.60 to 0.47)
IGs	24	0	1	1.09 (−0.05 to 2.22)	1.04 (−0.13 to 2.20)	−0.05 (−0.16 to 0.06)
Blasts	7	0	0	0.31 (−0.03 to 0.64)	0.31 (−0.03 to 0.64)	0.00 (0.00 to 0.00)
Plasma cells	1	0	0	0.04 (−0.05 to 0.14)	0.04 (−0.05 to 0.14)	0.00 (0.00 to 0.00)
nRBCs	9	0	0	0.39 (0.16 to 0.61)	0.39 (0.16 to 0.61)	0.00 (0.00 to 0.00)
Others	257	344	NA	−3.89 (−6.09 to −1.69)	NA	NA
**Mild Leukopenia (*n* = 10)**						
Neutrophils	1032	1005	1024	1.29 (−0.51 to 3.09)	−6.05 (−12.33 to 0.23)	−7.34 (−13.08 to −1.59)
Lymphocytes	570	553	679	0.57 (−2.60 to 3.74)	−9.65 (−14.56 to −4.74)	−10.22 (−16.71 to −3.73)
Monocytes	116	173	187	−2.40 (−4.06 to −0.74)	−4.48 (−8.49 to −0.47)	−2.08 (−6.07 to 1.91)
Eosinophils	39	56	55	−4.00 (−8.57 to 0.56)	−1.08 (−2.93 to 0.76)	−0.41 (−1.52 to 0.70)
Basophils	39	15	9	1.03 (0.40 to 1.67)	1.21 (0.50 to 1.91)	0.17 (−0.01 to 0.36)
IGs	65	36	25	1.25 (−0.15 to 2.65)	1.51 (0.02 to 3.01)	0.26 (−0.46 to 0.99)
Blasts	62	24	21	1.55 (0.38 to 2.72)	1.62 (0.46 to 2.77)	0.06 (−0.08 to 0.22)
Plasma cells	3	0	0	0.13 (−0.02 to 0.29)	0.13 (−0.02 to 0.29)	0.00 (0.00 to 0.00)
nRBCs	21	13	3	0.33 (0.06 to 0.59)	0.74 (0.07 to 1.41)	0.41 (−0.42 to 1.25)
Others	408	480	NA	−3.08 (−6.05 to −0.12)	NA	NA
**Moderate Leukopenia (*n* = 10)**						
Neutrophils	1125	1206	1485	−4.50 (−7.37 to −1.63)	−19.09 (−30.20 to −7.98)	−14.59 (−24.35 to −4.83)
Lymphocytes	266	268	346	0.70 (−2.92 to 4.32)	−4.99 (−12.40 to 2.42)	−5.70 (−11.29 to −0.11)
Monocytes	67	49	89	1.03 (0.22 to 1.83)	−1.03 (−3.44 to 1.39)	−2.05 (−4.80 to 0.69)
Eosinophils	8	11	25	−0.93 (−1.97 to 0.12)	−0.86 (−1.49 to −0.22)	−0.72 (−1.31 to −0.13)
Basophils	44	8	2	1.91 (1.24 to 2.58)	2.24 (1.34 to 3.14)	0.33 (−0.16 to 0.81)
IGs	47	21	20	1.29 (0.00 to 2.58)	1.29 (0.21 to 2.37)	0.00 (−0.72 to 0.72)
Blasts	10	0	0	0.49 (0.21 to 0.78)	0.49 (0.21 to 0.78)	0.00 (0.00 to 0.00)
Plasma cells	4	0	0	0.17 (−0.04 to 0.38)	0.17 (−0.04 to 0.38)	0.00 (0.00 to 0.00)
nRBCs	29	2	0	1.85 (−0.02 to 3.72)	1.95 (0.06 to 3.84)	0.10 (−0.05 to 0.25)
Others	378	413	NA	−2.81 (−8.16 to 2.55)	NA	NA
**Severe Leukopenia (*n* = 10)**						
Neutrophils	20	19	81	−2.45 (−11.04 to 6.14)	−9.92 (−24.97 to 5.14)	−7.47 (−16.61 to 1.67)
Lymphocytes	61	58	191	−2.55 (−9.45 to 4.36)	−47.74 (−71.45 to −24.03)	−45.19 (−72.82 to −17.56)
Monocytes	4	4	4	1.39 (−1.14 to 3.92)	0.15 (−3.25 to 3.56)	−1.24 (−3.11 to 0.64)
Eosinophils	1	0	7	−0.67 (−2.18 to 0.84)	−0.03 (−2.34 to 2.27)	−0.70 (−2.28 to 0.88)
Basophils	15	1	1	5.89 (2.42 to 9.36)	6.15 (2.64 to 9.65)	0.25 (−0.32 to 0.82)
IGs	6	0	0	1.92 (−0.31 to 4.16)	1.92 (−0.31 to 4.16)	0.00 (0.00 to 0.00)
Blasts	1	0	0	0.15 (−0.18 to 0.47)	0.15 (−0.18 to 0.47)	0.00 (0.00 to 0.00)
Plasma cells	2	0	0	0.86 (−0.75 to 2.47)	0.86 (−0.75 to 2.47)	0.00 (0.00 to 0.00)
nRBCs	16	0	0	9.07 (2.98 to 15.16)	9.07 (2.98 to 15.16)	0.00 (0.00 to 0.00)
Others	103	147	NA	−14.96 (−31.92 to 2.01)	NA	NA

Abbreviations: CI, confidence interval; IGs, immature granulocytes; NA, not available; nRBCs, nucleated red blood cells; WBC, white blood cells.

## Data Availability

The data presented in this study are available from the corresponding author on reasonable request.
